# Detection and Genetic Diversity of a Novel Water Buffalo Astrovirus Species Found in the Guangxi Province of China

**DOI:** 10.3389/fvets.2021.692193

**Published:** 2021-07-08

**Authors:** Qingli Fang, Mingyang Li, Haifeng Liu, Kuirong Chen, Yanjie Du, Chongli Wen, Yingyi Wei, Kang Ouyang, Zuzhang Wei, Ying Chen, Weijian Huang

**Affiliations:** ^1^Laboratory of Animal Infectious Diseases and Molecular Immunology, College of Animal Science and Technology, Guangxi University, Nanning, China; ^2^Scientific Research Center, Guilin Medicine University, Guilin, China; ^3^Breeding Farm of Water Buffalo, Guangxi Institute of Buffalo Chinese Academy of Agricultural Sciences, Nanning, China

**Keywords:** astrovirus, bovine, genetic diversity, Guangxi Province, neurotropic, water buffalo

## Abstract

Astroviruses (AstVs) are major causative agents of gastroenteritis and have been detected worldwide. Little is known about the prevalence of neurotropic AstVs in Chinese water buffaloes, but a novel species which is associated with encephalitis and meningitis has recently been found. In this study, based on nested RT-PCR, rapid amplification of the 3′-cDNA end (3′-RACE) and next-generation sequencing (NGS), we examined the infection of AstVs in water buffaloes in the Guangxi Province of China. The results showed that the AstV infection was found in 40% (6/15) of the farms examined, and the prevalence of AstV in their feces was 11% (33/297). In addition, two near-full-length and two complete open reading frame 2 (ORF2) genes of AstVs from fecal sources were sequenced. Phylogenetic analysis of the ORF2 sequences indicated three lineages of BufAstVs, BufAstV lineage 1 was close related to the BoAstV, lineage 2 was related to the BufAstVs, and lineage 3 was classified as novel AstVs, which had a close relationship with the neurotropic/neurovirulent AstVs strains found in bovine, ovine, and musks. Moreover, genomic a recombination between the BufAstV and BoAstV strains was identified. This is a novel study reporting the genetic diversity of BufAstV infection in China especially found the similar neurotropic strains from fecal sources of water buffaloes, and it also provides details of the epidemiology, genetic recombination, and interspecies transmission of BoAstV and BufAstV in water buffaloes from the Guangxi Province of China.

## Introduction

Astroviruses (AstVs) are non-enveloped, single-stranded positive-sense RNA viruses (1). They are 6.4–7.7 kb in length and usually contain several consecutive and partially overlapping open reading frames (ORFs): ORF1a, ORF1b, ORF2, and ORFX (also termed ORF2b) (2, 3). Both ORF1a and ORF1b encode non-structural proteins. ORF2 is expressed from the subgenomic RNA and encodes a capsid structural protein (2). AstVs have a broad range of hosts and are classified with different genotypes according to the similarity of amino acid sequence of the capsid precursor protein encoded by ORF2 (4). AstVs are generally considered to be the major causative agents for diarrhea in children and other immunodeficient hosts (5). However, in recent years, novel AstVs have been found that can cause extra-gastrointestinal diseases, such as hepatitis, nephritis, meningitis, and encephalitis as well as and other neurological symptoms in humans and several animal hosts (6).

Bovine AstV (BoAstV) was first discovered in calves with diarrhea in 1978 (7). However, the pathogenicity of BoAstV is not clear. Recently, novel neurotropic/neurovirulent BoAstV strains, such as BoAstV NeuroS1 (KF233994.1), BoAstV CH13 (NC 024498.1), BoAstV KagoshimaSR28 (LC341267), and BoAstV BH89/14 (LN879482.1) have been identified from the United States, Switzerland, Japan, Germany, Italy, and the United Kingdom. These can infect the central nervous system (CNS) and cause meningitis and encephalitis, subsequently leading to serious neurological signs (8, 9). In addition, the interspecies transmission and recombination cases of AstVs deserve attention. The frequency of genetic recombination of ORF2 between different species may be the main reason for interspecies transmission of these viruses, particularly between similar genetic hosts (e.g., ovine and bovine, wild boars and swine as well as primates and humans) because the species barrier of AstVs is not particularly strong (6).

The Guangxi Province is one of the largest regions where water buffaloes are bred in China. The prevalence and genetic diversity of water buffalo AstV (BufAstV) in China are still poorly documented. In particular, very little is known regarding the prevalence of different neurotropic strains of AstVs in China. Therefore, in this study, 297 water buffalo fecal samples from 15 different scale farms in five regions of the Guangxi Province were examined for AstVs. This study reports the genetic diversity of BufAstV infection from water buffalo feces and also provides evidence of genetic recombination of these viruses.

## Materials and Methods

### Sample Collection

A total of 297 fecal and 40 serum samples were collected from water buffaloes reared in 15 different farms in the Nanning, Guigang, Beihai, Hengxian, and Linshan regions of Guangxi Province in 2019 (Table 1). Fecal samples were collected in autoclaved centrifuge tubes and dispersed as 10% suspensions in phosphate buffered saline (PBS) (pH 7.2), and then centrifuged for 10 min at 12,000 rpm and 4°C. Serum samples were also collected and centrifuged for 10 min at 2,000 rpm in 4°C. The supernatants from fecal and serum samples were used to extract viral nucleic acids and these were stored at −80°C.

**Table 1:** Details of sample information and results.

**Sampling location**	**Date**	**Number of samples and the age**	**No. of positive**	**Positive rate**	**Partial ORF1b clone names and GenBank accession numbers**
Nanning A	2019.4	10 (>150 days)	6	80%	NNA-6 (MT492426), NNA-7 (MT492425), NNA-12 (MT492423), NNA-14 (MT492439), NNA-15 (MT492440), NNA-17 (MT492438), NNA-11 (MT492424), NNA-13 (MT492422)
		10 (<150 days)	10		
Nanning B	2019.4	10 (>150 days)	0	0	
		10 (<150 days)			
Nanning C	2019.4	20 (<150 days)	8	40%	NNC-286 (MT492437), NNC-296 (MT492436)
Nanning D	2019.5	20 (<150 days)	2	10%	NND-S2 (MT492435), NND-S16 (MT492434)
Nanning E	2019.5	20 (>150 days)	0	0	
Beihai A	2019.6	10 (>150 days)	0	0	0
		10 (<150 days)			
Beihai B	2019.6	10 (>150 days)	0	5%	BH-C14 (MT492433)
		10 (<150 days)	1		
Beihai C	2019.6	10 (>150 days)	0	5%	BH-C22 (MT492432)
		10 (<150 days)	1		
Beihai D	2019.6	9 (>150 days)	0	0	
		9 (<150 days)			
Guigang A	2019.10	20 (>150 days)	0	0	
Guigang B	2019.10	25 (>150 days)	0	0	
Hengxian A	2019.9	29 (>150 days)	0	0	
Hengxian B	2019.9	24 (<150 days)	5	20%	HX-1 (MT492431), HX-3 (MT492430), HX-4 (MT492429), HX-5 (MT492428), HX-6 (MT492427)
Lingshan A	2019.8	21 (<150 days)	0	0	
Total samples: 297		153 (>150 days)	6	4%	Total positive rate: 11%
		144 (<150 days)	27	18%	

### RNA Extraction and RT-PCR

RNA was extracted from supernatants of rectal swabs using an RNAiso Plus kit (Takara Bio, Inc., Dalian, China) following the manufacturer's protocols. The first-strand cDNA was synthesized using a PrimeScript II 1st strand cDNA synthesis kit (Takara Bio, Inc.). The partial RNA-dependent RNA polymerase (RdRp), which was gene specific for AstVs, was amplified using the nested PCR method referred by Chu et al. (10) (Supplementary Table 1), and this was sequenced as described previously (1).

### 3′-RACE and Next-Generation Sequencing

The ORF2 gene of AstVs was amplified using a 3′-RACE PCR kit (Takara, Bio, Inc.) following the manufacturer's protocols. Specific primers were designed according to the RdRp sequences, and these are listed in Supplementary Table 1. Next-generation sequencing (NGS) was performed to obtain the full-length genome of AstVs. The first- and second-strand cDNAs were synthesized using First-Strand Synthesis Enzyme Mix and Second-Strand Synthesis Enzyme Mix (ABclonal, Wuhan, China), respectively. A cDNA library was constructed using a Rapid DNA Lib Prep Kit (Illumina, CA, USA). Bridge PCR was performed using a TruSeq PE Cluster Kit (Illumina). Sequencing was performed on an Illumina Novaseq6000 instrument using a TruSeq SBS Kit v3 (Illumina, CA, USA), and the length of each read generated by sequencing was between 100 and 150 bp. After a comprehensive quality control procedure to filter data by using Fastp software, the sequence data were assembled by *de-novo* methods, which employed SPAdes and MEGAHIT software packages.

### Phylogenetic and Genome Analysis

All the obtained sequences were uploaded to the GenBank in this study and aligned against other AstV reference sequences found in NCBI (https://blast.ncbi.nlm.nih.gov/Blast.cgi) by using the ClustalW (1.6) method in the MEGA 7.0 software. The same software was used to reconstruct phylogenetic trees from evolutionary distances using the neighbor-joining (NJ) method with p-distances for nucleotide sequences and 1,000 replicates for the bootstrap test, which evaluated their clustering stabilities. The p-distance analysis was conducted with MEGA 7.0 software. Standard error estimate(s) are shown above the diagonal and were obtained by a bootstrap procedure using 1,000 replicates. Analyses were conducted using the Maximum Composite Likelihood model. The accession numbers of the nucleotide sequences obtained in this study and reference sequences are shown in Supplementary Table 2.

### Recombinant Analysis

The full-length of ORF2 sequences of AstVs were screened for recombinant signals using the RDP4 recombination program v.4.39 by RDP, GENOCONV, BootScan, MaxChi, Chimaera, and Siscan recombinant algorithm methods. When there were at least three signals with *p*-values < 0.05, these were considered potential recombinant events. These were then further subjected to similarity plots and BootScan analysis using the Kimura (two parameter) method to the NJ model with 1,000 bootstrap replicates using SimPlot v3.5.1. The phylogenetic trees of different portions of recombinant regions between the breakpoints were constructed by the NJ method to analyze the potential recombinant sequences at both ends of the breakpoints as described above.

## Results

### The Prevalence of BufAstV in Guangxi Province

In this study, all the fecal samples were obtained from water buffaloes that had no significant clinical symptoms. The AstV-infection was found in 40% (6/15) of the farms detected, and the AstV-positive rate of feces was 11% (33/297), while the AstV-positive rate of serum was 0. The positive rate of calves (<150 days old) was higher than the positive rate of adult water buffaloes. All the relevant information related to the samples is shown in Table 1.

### Phylogenetic and Genome Analysis

Two near-full-length genes of AstVs (BufAstV-NNA-12, GenBank accession: MT499771; BufAstV-NNA-14 GenBank accession: MT499772) were obtained by NGS in this study. The length of BufAstV-NNA-12 and BufAstV-NNA-14 was 6,230 nt and 6,406 nt, respectively, and both contained the full-length ORF1ab, ORF2, and complete 3′-untranslated regions (3′-UTR) consisting of a poly A tail and the near-full-length 5′-UTR. The similarity of ORF1ab between BufAstV-NNA-12 and BufAstV-NNA-14 was 52.9%. Except for BufAstV-NNA-12 and BufAstV-NNA-14, two full-length sequences of ORF2 named BufAstV-NND-s2 (Gen-Bank accession: MT521688) and BufAstV-NNA-17 (GenBank accession: MT521687) were obtained by 3′-RACE. The nucleotide and amino acid identities between the four ORF2s in this study were 41.1–57.5% and 20.7–47%, respectively. The highest nucleotide and amino acid identities between BufAstV-NND-s2 and BufAstV-NNA-12 were only 57.4 and 47%, respectively, and the BufAstV-NNA-14 had a much lower identity compared with the other ORF2 sequences.

**Figure 1:**
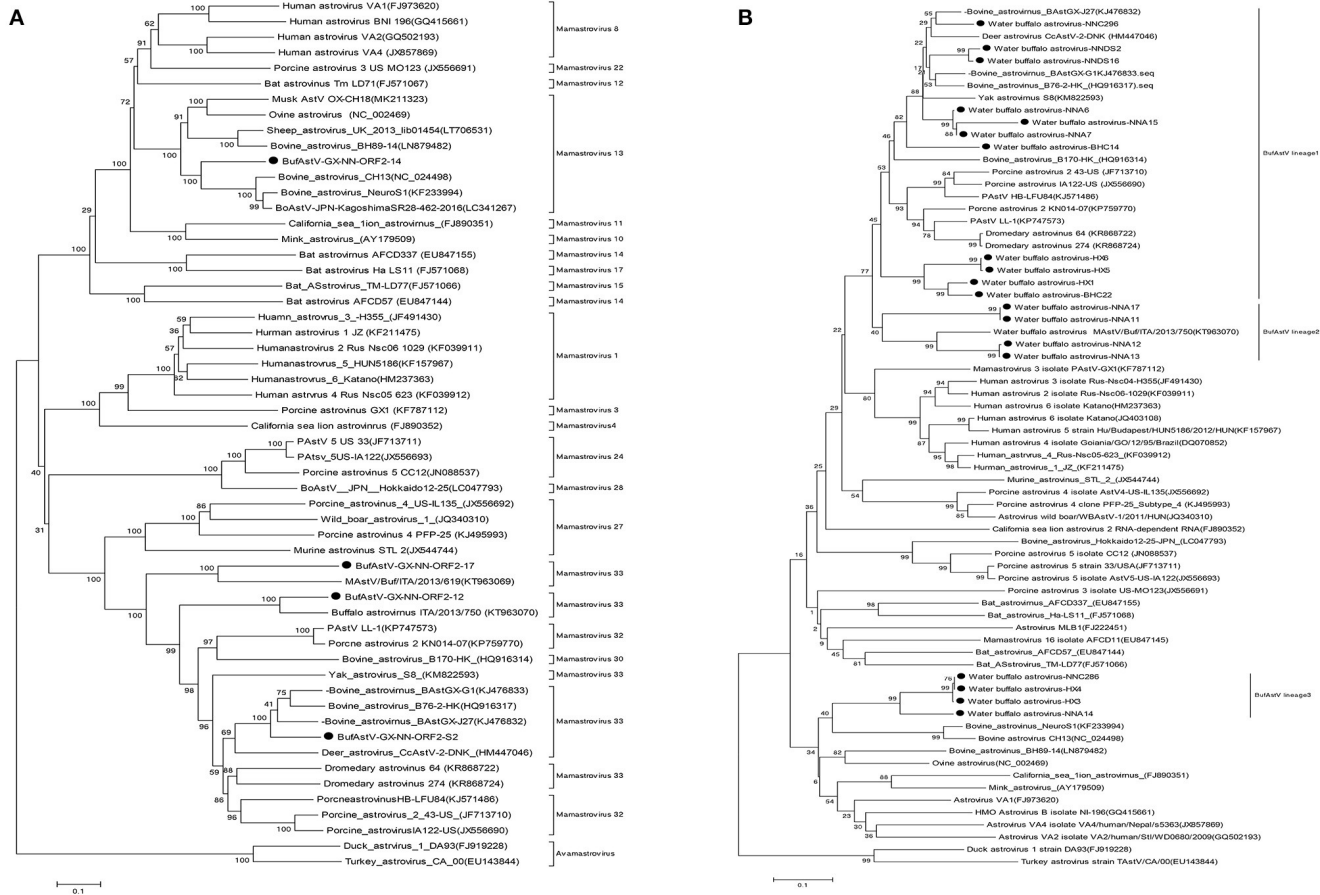
The neighbor-joining phylogenetic trees of the full-length ORF2 **(A)** and partial ORF1b genes **(B)** of AstVs with *p*-distances for nucleotide sequences and 1,000 replicates were used for the bootstrap test. The black dots indicate the sequences from this study.

Based on the NJ phylogenetic tree of ORF2, BufAstV-NNA-14 was seen to be similar to the bovine neurotropic strains such as BoAstV/JPN/KagoshimaSR28-462 (GenBank: LC341267) and BoAstV CH13 (GenBank: NC_024498), which cause meningitis and encephalitis. They all belong to the Mamastrovirus 13 clade, and they contain the major neurotropic AstV strains from ruminants (Figure 1A). On the other hand, BufAstV-NND-s2 clustered in the Mamastrovirus 33 that was closely related with BoAstV B76/HK (GenBank: HQ916317) and BoAstV GX/G1 (GenBank: KJ476833). The identity with the BoAstV B76/HK strain was much higher than the identity with other BufAstV strains. The p-distance of ORF2 between BufAstV-NND-s2 and BoAstV B76-2/HK was only 0.181, suggesting that the former could be classified as BoAstV, although it was isolated from water buffalo feces. In addition, the isolated strains, BufAstV-NNA-17 and BufAstV-NNA-12, were clustered with other BufAstVs strains, such as MAstV/Buf/ITA/2013/750 (GenBank: KT963070) and MAstV/Buf/ITA/2013/619 (GenBank: KT963069).

NJ phylogenetic analysis was performed using the nucleotide sequences from the 3′-terminal conserved regions of the partial ORF1b gene segments. These were amplified from the ORF1b (RdRp) primers by nested PCR (Supplementary Table 1). The results of phylogenetic analysis were consistent with those obtained from the complete ORF2 gene. In this study, three different genetic lineages of BufAstV were found in the Guangxi Province: BufAstV lineage 1 was found to be closely related to the BoAstV strains, lineage 2 was related to the BufAstVs strains, and lineage 3 was classified as possible neurotropic AstVs, which had a close relationship with the neurotropic/neurovirulent AstVs strains found in bovine and ovine lineages as well as in musks (Figure 1B).

### Recombinant Analysis Between BoAstV and BufAstV

**Figure 2:**
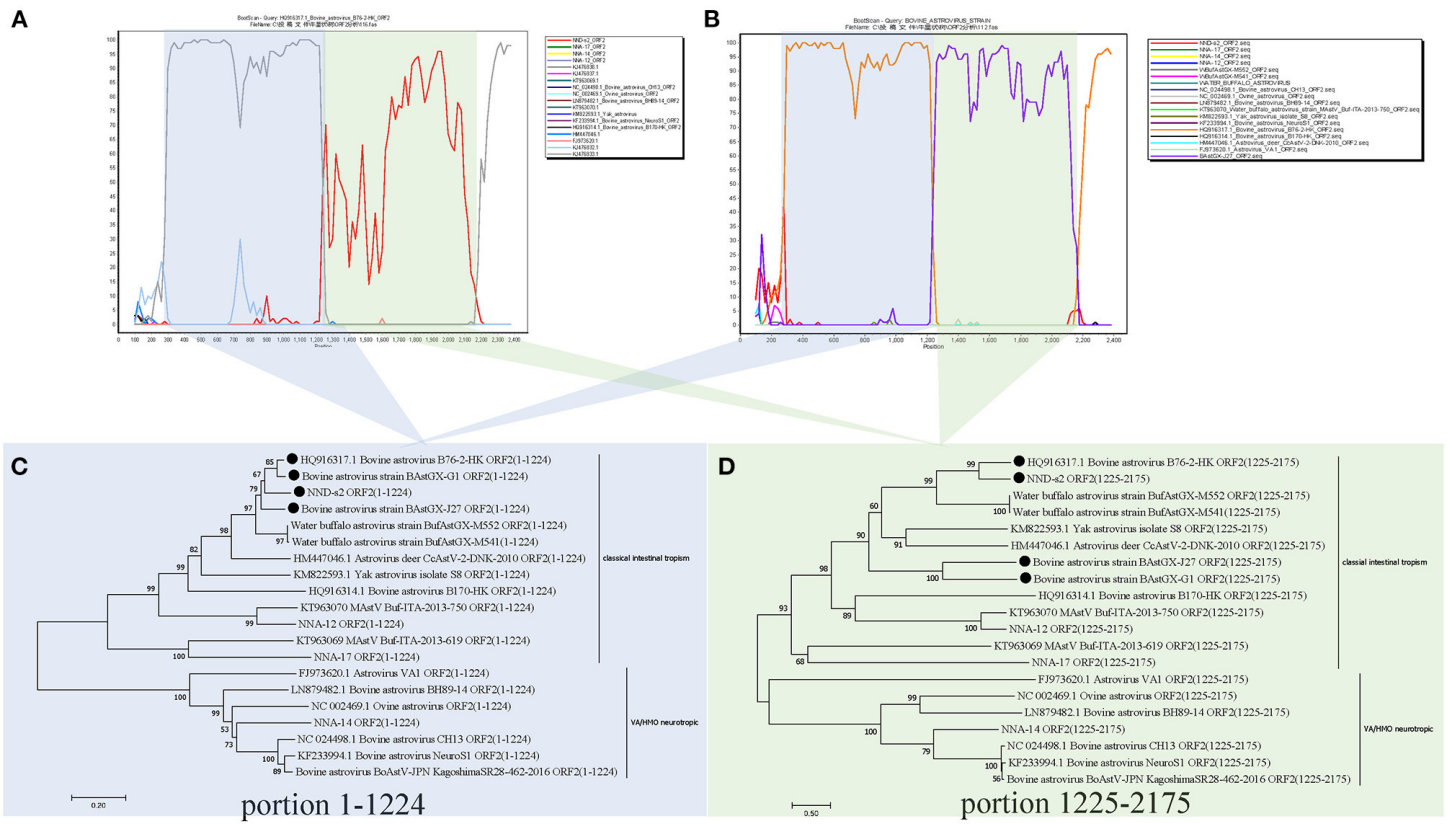
BootScan recombination analysis based on the ORF2 gene of bovine Astrovirus B76-2-HK **(A)** and bovine Astrovirus GX1 **(B)** using the two-parameter (Kimura) distance and the neighbor-joining models were performed using 1,000 bootstrap replicates. The NJ phylogenetic trees of 1–1,224 nt **(C)** and 1,225–2,175 nt **(D)** of ORF2. In the clade of intestinal tropism AstVs, different topologies of phylogenetic trees are shown between portions 1–1,224 and 1,225–2,175. The construction was performed as described in the Results section. The black dots indicate the recombinant strains used in this study.

The full-length ORF2 sequences of AstVs were screened for recombinant signals by using RDP4 and SimPlot software packages. Strong recombination signals between different BoAstV sequences were found in RDP4, which were then reconfirmed with SimPlot. The analysis results showed that the sequences of BoAstV B76-2-HK was partially recombined with BoAstV GX1 and BufAstV-NND-s2 as well as with BoAstV GX1 and BoAstV GX-J27 in their original ORF2 sequences (Figure 2). The recombinant breakpoint positions of these sequences were 1,225 and 2,175 nt, and based on these positions, the ORF2 was divided into two recombinant regions (1–1,224 and 1,225–2,175), which were found to be consistent with the conserved and hypervariable regions of AstVs ORF2 (11). Two phylogenetic trees were constructed for separating these recombinant regions of the ORF2 of AstVs at the breakpoint positions, respectively. The results confirmed that different recombinant regions had inconsistent topologies (Figure 2).

## Discussion

BoAstV was first discovered in calves with diarrhea in 1978 and was long been considered to be avirulent until neurotropic strains of BoAstV were discovered (4). In the present study, we reported the infection of AstVs in water buffalo herds from 15 different scale farms in five regions of Guangxi Province. Based on the RT-PCR, 3′-RACE and NGS technology, we obtained the partial RdRp genes, complete ORF2 genes, and the near-full-length genomes of AstV from positive samples. The phylogenetic analysis found the AstV strains could divide three different genetic lineages with large differences: BufAstV lineages 1, 2, and 3. It is worth noting that BufAstV lineage 3 (representative strain: BufAstV NNA-14) was clustered in Mamastrovirus 13, which had a longer genetic distance compared with the other BufAstV strains, and this clade contained the major neurotropic AstV strains of ruminants like BoAstV, ovine/sheep astrovirus (OvAstV), and musk AstV. This is the first report of a novel BufAstV with a Mamastrovirus 13 genotype has been found in China.

In this study, AstV infection was found in 40% (6/15) of the farms sampled, and the BufAstV-positive rate in collected fecal samples was 11% (33/297). No evidence was found to show that viral infection caused AstVs viremia. The positive rate of BufAstV was much lower than that in the previous study (36.8%) (1), mainly because the samples were collected from buffaloes of a different age group. In the previous study, fecal samples were collected from calves, while in this study, samples were collected from buffaloes of all ages. Significantly, in this study, the positive rate of calves (<150 days old) was higher than the positive rate of adult water buffaloes. These findings also indicated that the infection rate of BufAstV was higher in calves. In addition, the buffaloes collected in this study did not exhibit obvious symptoms of diarrhea. Whether the intestinal AstV is pathogenic in ruminants still needs further investigation. The nested RT-PCR detection method used was carried out with reference to degenerate primers of nested RT-PCR designed by Chu et al. (10). This method was originally designed to detect Bat AstV, which was later found to be specific for different Mamastrovirus genotypes, such as porcine AstV, BoAstV, primate AstV, tiger AstV, and canine AstV (12–16). However, there have been no recent studies using this method to detect of neurotropic AstVs strains. Although we detected the BufAstV NNA-14 neurotropic AstV in feces and brain tissue (data not shown) by using the degenerate primers of nested RT-PCR, the detection rate of the BufAstV lineage 3 was much lower than that for other lineages in this study. The specificity of these primers for BufAstV lineage 3-related strains was not clear, leading to deviations in the positive rate of this lineage 3, and it is envisaged that the actual infection might be higher than that currently reported.

AstVs have the characteristics of co-infection and possible interspecies transmission, which greatly increases the probability of genetic recombination and mutations between different AstVs strain, particularity in hosts with a greater level of interaction or host genetic similarity (e.g., bovine species and water buffalo) (6). However, little is known about the genetic diversity of BufAstVs. Based on the phylogenetic analysis, the identity of isolated strains in the BufAstV lineage 1 had a closer phylogenetic distance with BoAstVs compared with other BufAstV strains, suggesting that these strains might be classified into BoAstVs. These results indicated the possibility of interspecies transmission of BoAstV in water buffalo, suggesting the susceptibility of water buffalo to both BoAstVs and BufAstVs. Because of the close relationship between water buffalo and cattle, it might be appropriate to consider the presently identified BoAstVs and BufAstVs as different genotypes of the same species of AstVs despite having different hosts. This was similar to the situation with feline and cheetah AstVs (17). In addition, the genetic recombination between BoAstV and BufAstV strains was found in this study. Considering the phylogenetic distance with BoAstVs compared with other BufAstV strains, we speculated that these recombination events were not unusual in bovine species or in buffalo. Interestingly, the recombination breakpoint position was located at 1,225, which divided the ORF2 into two regions. These regions translated the capsid protein, which subsequently influenced the properties related to viral virulence, tropism, and epitope content of the resultant virus (11). Recombination in these regions might generate the diversity of capsid proteins and enable the AstVs to escape host immunity and expand tropism or host range.

Generally, AstVs are considered to be enteroviruses, and many of the infections seen in healthy children and adults tend to be asymptomatic (5). Recently, AstVs have been found to be associated with the diseases of the CNS in humans or other animal species (18). However, no similar cases were reported in China before. According to the latest research by Kuchler et al., their immunohistochemistry (IHC) analysis showed positive AstV- immunostaining in various brain regions from a sheep that died of encephalitis in 1992. The NGS and phylogenetic analysis found a close genetic relationship between this OvAstV sequence and the BoAstV-CH15/OvAstV-CH16 strains (9). This finding suggested that the neurotropic AstVs have been present for more than two decades and was only identified due to the recent development of NGS technology. Therefore, infections by neurotropic AstVs may well have been occurred in China but remained undetected for some time and this requires to be further investigated.

The phylogenetic analysis revealed the clustering of BufAstV-NNA14 strain into Mamastrovirus 13 genotype, which includes the major neurotropic AstVs from ruminants, indicates that these strains have the same origin (8). Also, the neurotropic AstV strains found in different hosts, such as human, pig, ovine, bovine, and mink, were divided into a big clade in the phylogenetic trees, suggesting a close phylogenetic evolution relationship and the possibility of the interspecies transmission of neurotropic AstVs between human and animals (19). In addition, compared with other lineages of AstVs, the higher homology of neurotropic AstVs in Mamastrovirus 13 might also be more proned to interspecies transmission events. Moreover, the similar neurotropic AstV was first to reported in the feces collected in this study. This was similar to the results of previous studies by Kauer et al. on the detection of neurotropic BoAstVCH13/NeuroS1 in feces (20). These studies indicated that the neurotropic AstV might also cause intestinal infections in a similar way to enteric AstV, and they might have a similar fecal–oral transmission mechanism. However, neurotropic strains might have some special mechanisms that enable them to go through the gastrointestinal tract and invade the CNS, thus causing serious damage such as encephalitis. This will prompt continued surveillance of the development of clinical symptoms in water buffalo herds, which are infected with neurotropic BufAstV-NNA-14.

In this study, BufAstV NNA-14 is a novel BufAstV with a Mamastrovirus 13 genotype, which contains the most of the neurotropic/neurovirulent AstV strains from ruminants such as BoAstV NeuroS1 (KF233994.1), BoAstV CH13 (NC 024498.1), BoAstV BH89/14 (LN879482.1), ovine AstV (NC_002469), and musk AstV OX-CH18 (MK211323). These strains are neurotropic and can infect CNS cause viral meningitis and encephalitis in previous reports (8, 18, 21, 22). This result suggested that BufAstV NNA-14 is similar neurotropic AstV strain and may have the ability to infect the CNS cause encephalitis in water buffalo. We conducted a return visit to the farm where BufAstV NNA-14 was detected and found the unexplained neurological symptoms that led to the death of a calf. We performed necropsied and IHC analysis to confirm whether the BufAstV-NNA-14 strain existed in CNS (data not shown). Unfortunately, histopathology analysis found that was not characteristic for a non-suppurative encephalitis, and the strongly viral antigens label in brain sections was not been found, suggested neither histopathology lesions nor IHC analysis could convincingly prove the viral encephalitis and viral infection in this case. This might be because the virus had not yet infected the brain or the clearance of viral components by the immune reaction system, suggesting that BufAstV NNA-14 infection was related to individual differences, the immune response of the host, and sensitivity of the degenerate primers used in the nested RT-PCR method. We also analyzed other organs including the lungs, brainstem, spleen, liver, and intestinal lymph nodes by nested RT-PCR; however, the AstV-positive was only present in the intestinal tissues. Although no obvious clinical signs were found, considering the uncertain pathogenicity of neurotropic BoAstV, further research should continue to focus on clinical observations of water buffalo herds infected with neurotropic BufAstV-NNA-14.

## Data Availability Statement

The datasets presented in this study can be found in online repositories. The names of the repository/repositories and accession number(s) can be found in the article/Supplementary Material.

## Ethics Statement

The animal study was reviewed and approved by Animal Care & Welfare Committee of Guangxi University (grant no. GXU2018-044). Written informed consent was obtained from the owners for the participation of their animals in this study.

## Author Contributions

WH, ZW, and YC: conceived and designed the experiments. QF, ML, HL, KC, and YD: experiments performed. QF: formal analysis and writing, original draft preparation. ML and HL: samples collection. CW and ML: clinical anatomy. QF, YW, and KO: writing, review, and editing. WH: project administration and funding acquisition. All authors contributed to the article and approved the submitted version.

## Conflict of Interest

The authors declare that the research was conducted in the absence of any commercial or financial relationships that could be construed as a potential conflict of interest.
