# Supplementary material for: Detection and Genetic Diversity of a Novel Water Buffalo Astrovirus Species Found in the Guangxi Province of China
Source: Front Vet Sci. 2021 Jul 8;8:692193. doi: 10.3389/fvets.2021.692193 (PMC8295894; doi:10.3389/fvets.2021.692193)
Supplement: Supplementary file 1 [file Data_Sheet_1.docx]

Supplementary Table 1： Primer information

| Target  gene | NameofPrimer | Primersequences(5'→3') | Product size/bp | Reference |
| --- | --- | --- | --- | --- |
| NNA-12 | GSP1a | GAATAAGAACAGTGGCCACGAC | 2322 | Inthisstudy |
|  | GSP1b | GATAGGTATGTCCTCATGCC |  |  |
| NNA-14 | GSP2a | TTGCGAGCCATGTATGAGTGGT | 2289 | Inthisstudy |
|  | GSP2b | TTGTGGTAATCCCTCAGGCCAA |  |  |
| NNA-17 | GSP3a | AAGGCATGCGTGACTGGTATGT | 2217 | Inthisstudy |
|  | GSP3b | CGATCGTTTATGGAGACGATCG |  |  |
| NND-s2 | GSP4a | CCTTCAGGACAGATTTCCACCA | 2271 | Inthisstudy |
|  | GSP4b | CTTTGAGTTCGCCTTCCTGAAC |  |  |
|  | Innerprimer | TACCGTCGTTCCACTAGTGATTT |  |  |
|  | Outerprimer | CGCGGATCCTCCACTAGTGATTTCACTATAGG |  |  |
| Partial ORF1b  (RdRp) | PanAV-F11 | GARTTYGATTGGRCKCGKTAYGA | 422 | ([Chu et al., 2008](#_ENREF_5)) |
|  | PanAV-F12 | GARTTYGATTGGRCKAGGTAYGA |  |  |
|  | PanAV-F21 | CGKTAYGATGGKACKATNCC |  |  |
|  | PanAV-F22 | AGGTAYGATGGKACKATNCC |  |  |
|  | PanAV-R1 | GGATTKACCCACATNCCRYY |  |  |
| BufAstV-NNA-14 partial ORF2 | F | gccatggctgatatcggatccCCTTTTTATCTGGCCTCATGTTATG | 804bp | In this study |
|  | R | ttgtcgacggagctcgaattcGTCGCTGGCAAATCGGTAGC |  |  |
| Rabies Virus | F | ACGCTTAACAACMARAYCAA | 495bp | (Xian et al., 2018) |
|  | R | CCGACTAAAGATGCATGCTC |  |  |
| Japanese encephalitis | F | ACGAGACCGATCAATCGCT | 220bp | (Loan et al., 2015) |
|  | R | CTTTGTGGACGATCTTCGCT |  |  |
| Pseudorabies virus | F | CGTCACCGAGGTCCCGAGTCCCT | 1263bp | (Wang et al., 2015) |
|  | R | GGAGCACAGCACGCAGAGCCAGAC |  |  |
| Bovine herpesviruses | F | CGGCCACGACGCTGACGA | 575bp | Esteves et al., 2008) |
|  | R | CGCCGCCGAGTACTACCC |  |  |

Supplementary table 2： The Information regarding the reference sequences in this study

| strain | Genbank | Strain | Genbank |
| --- | --- | --- | --- |
| YakastrovirusS8 | KM822593 | Human astrovirus 6 Katano | HM237363 |
| Wildboarastrovirus 1 | JQ340310 | Human astrovirus 5 HUN5186 | KF157967 |
| TurkeyastrovirusCA00 | EU143844 | Human astrovirus VA4 | JX857869 |
| Porcine astrovirus HBLFU84 | KJ571486 | Human astrovirus VA2 | GQ502193 |
| Porcine astrovirus 2 KN01407 | KP759770 | Human astrovirus VA1 | FJ973620 |
| Porcineastrovirus IA122US | JX556690 | Human astrovirus BNI196 | GQ415661 |
| Porcineastrovirus 3 USMO123 | JX556691 | Humanastrovirus 4 Goiania | DQ070852 |
| Human astrovirus 5 DL030 | JF713710 | Human astrovirus 2 RusNsc061029 | KF039911 |
| Porcine astrovirusGX1 | KF787112 | Humanastrovirus 3 H355 | JF491430 |
| Porcine astrovirus 5 CC12 | JN088537 | Duck astrovirus 1 DA93 | FJ919228 |
| Porcineastrovirus 4 USIL135 | JX556692 | Dromedary astrovirus 274 | KR868724 |
| Porcine astrovirus 4 PFP-25 | KJ495993 | Dromedary astrovirus 64 | KR868722 |
| Porcineastrovirus 5USIA122 | JX556693 | Deer astrovirus CcAstV2DNK | HM447046 |
| Porcineastrovirus LL1 | KP747573 | California sea lion astrovirus | FJ890352 |
| Porcineastrovirus 5US33 | JF713711 | Bovine astrovirus Hokkaido12-25 | LC047793 |
| Ovine astrovirus | NC002469 | Bovine astrovirus B170HK | HQ916314 |
| Murine astrovirus STL 2 | JX544744 | Bovine astrovirus BAstGXJ27 | KJ476832 |
| Bovine astrovirus BH8914 | LN879482 | Bovine astrovirus BAstGXG1 | KJ476833 |
| Mink astrovirus | AY179509 | Bovine astrovirus B762HK | HQ916317 |
| Human astrovirus 1 JZ | KF211475 | Bat astrovirus HaLS11 | FJ571068 |
| Bat Astrovirus TMLD77 | FJ571066 | Bat astrovirus AFCD57 | EU847144 |
| Bovine astrovirus CH13 | NC024498 | Bat astrovirus AFCD337 | EU847155 |
| Bovine astrovirus NeuroS1 | KF233994 | Bat astrovirus TmLD71 | FJ571067 |
| California sea 1ion astrovirus | FJ890351 | water buffalo astrovirus Italy 2013-750 | KT963070 |
| Human astrovirus 4 Rus Nsc05 623 | KF039912 | Bovine astrovirus CH13 | NC024498 |
| water buffalo astrovirus Italy2013-619 | KT963069 | Bovine astrovirus KagoshimaSR28-462 | LC341267 |
| Musk AstV OX-CH18 | MK211323 | Sheep_astrovirus_UK_2013_lib01454 | LT706531 |
